# In vivo demonstration of globotriaosylceramide brain accumulation in Fabry Disease using MR Relaxometry

**DOI:** 10.1007/s00234-024-03380-5

**Published:** 2024-05-21

**Authors:** Giuseppe Pontillo, Mario Tranfa, Alessandra Scaravilli, Serena Monti, Ivana Capuano, Eleonora Riccio, Manuela Rizzo, Arturo Brunetti, Giuseppe Palma, Antonio Pisani, Sirio Cocozza

**Affiliations:** 1 Department of Advanced Biomedical Sciences, University of Naples “Federico II”, Via Pansini 5, 80131 Naples, Italy; 2 Institute of Biostructure and Bioimaging, National Research Council, Naples, Italy; 3 Department of Public Health, University of Naples “Federico II”, Naples, Italy; 4 Institute for Biomedical Research and Innovation, National Research Council, Palermo, Italy; 5 Institute of Nanotechnology, National Research Council, Lecce, Italy

**Keywords:** Brain, Fabry Disease, Magnetic Resonance Imaging, Relaxometry

## Abstract

**Purpose:**

How to measure brain globotriaosylceramide (Gb3) accumulation in Fabry Disease (FD) patients in-vivo is still an open challenge. The objective of this study is to provide a quantitative, non-invasive demonstration of this phenomenon using quantitative MRI (qMRI).

**Methods:**

In this retrospective, monocentric cross-sectional study conducted from November 2015 to July 2018, FD patients and healthy controls (HC) underwent an MRI scan with a relaxometry protocol to compute longitudinal relaxation rate (R1) maps to evaluate gray (GM) and white matter (WM) lipid accumulation. In a subgroup of 22 FD patients, clinical (FAbry STabilization indEX -FASTEX- score) and biochemical (residual α-galactosidase activity) variables were correlated with MRI data. Quantitative maps were analyzed at both global (“bulk” analysis) and regional (“voxel-wise” analysis) levels.

**Results:**

Data were obtained from 42 FD patients (mean age = 42.4 ± 12.9, M/F = 16/26) and 49 HC (mean age = 42.3 ± 16.3, M/F = 28/21). Compared to HC, FD patients showed a widespread increase in R1 values encompassing both GM (*p*_FWE_ = 0.02) and WM (*p*_FWE_ = 0.02) structures. While no correlations were found between increased R1 values and FASTEX score, a significant negative correlation emerged between residual enzymatic activity levels and R1 values in GM (*r* = -0.57, *p* = 0.008) and WM (*r* = -0.49, *p* = 0.03).

**Conclusions:**

We demonstrated the feasibility and clinical relevance of non-invasively assessing cerebral Gb3 accumulation in FD using MRI. R1 mapping might be used as an in-vivo quantitative neuroimaging biomarker in FD patients.

## Introduction

Fabry Disease (FD) is a multiorgan X-linked lysosomal storage disorder caused by mutation in the GLA gene (Xq22.1), encoding for the α-galactosidase A (α-GalA) enzyme [1]. Its defective activity leads to intracellular accumulation of glycosphingolipids, mainly globotriaosylceramide (Gb3), in multiple tissues and organs, with prominent involvement of kidney, heart and Central Nervous System (CNS) [2]. Although cerebrovascular events are among the most relevant complications of FD, CNS involvement in this condition goes far beyond cerebrovascular accidents, including neuropathic pain, cochleovestibular dysfunction, a various degree of motor and cognitive impairment and psychiatric symptoms [3–5]. Nevertheless, the exact pathophysiological mechanisms underlying CNS involvement in FD are not completely known. Indeed, along with Gb3 deposition occurring in endothelium and vascular smooth muscle cells, direct glial and neuronal deposition have also been hypothesized as possible mechanisms for primary neurodegeneration and atrophy [6]. In particular, pathological studies have demonstrated the presence of neuronal “ballooning” reflecting lipid accumulation in different brain areas, including hippocampus, amygdala, deep gray matter (GM) and fronto-parietal cortex [7, 8].

MRI is the reference imaging technique to evaluate possible brain damage in FD in vivo, allowing for a proper qualitative estimation of the pattern and extension of brain alterations, especially at the level of the white matter (WM), in patients with or without clinical evidence of neurological impairment [9]. Different conventional MRI signs in FD have been described in FD, including the pulvinar sign, the dolichoectasia of the basilar artery, and the occurrence of WM lesions [9]. Nonetheless, all these signs are either very uncommon (as in the case of the pulvinar sign [10]), or suffer of a lack specificity, with basilar artery dolichoectasia that are commonly observed in young patients with other uncommon causes of stroke or WM lesions not showing any peculiar appearance or spatial distribution [9]. On the other hand, different advanced MRI techniques have been recently applied in FD, to better elucidate pathophysiological mechanisms underlying CNS involvement in this disorder. These include, but are not limited to, volumetric MR analysis, to investigate the possibility of brain tissue volume loss, as well as diffusion and functional MRI, techniques used to assess structural and functional connectivity changes that might occur in these patients [9]. Among these, quantitative MRI (qMRI) is particularly noteworthy, providing an accurate characterization of brain tissue properties by measuring physical parameters intrinsically related to tissue microstructure [11]. This approach allows the quantification not only of iron accumulation via Quantitative Susceptibility Mapping (QSM) [10, 12], but also of lipid deposition with the computation of longitudinal relaxation rate (R1) maps. These maps allows for the evaluation of the degree of hydration of a specific tissue: an increase in concentration of high weight molecules, such as lipids, modifies the degree of hydration, leading to an increase in R1 values [11]. Under specific circumstances, such as FD, this qMRI metric can be therefore used as a proxy of lipid deposition, thus bearing the potential to directly characterize the primary pathophysiological mechanism of FD. In-vivo quantification of Gb3 accumulation using these qMRI techniques has already been applied for other organs in FD. In particular, T1 mapping has found extensive application in cardiac imaging, where lower T1 values supposedly reflect myocardial Gb3 deposition, [13] and reports exist exploring a possible similar role in kidney imaging [14].

Objective of this study is to provide a quantitative, accurate, in-vivo and non-invasive demonstration of Gb3 accumulation in FD using qMRI, with the aim of further expanding the current knowledge about the pathophysiology of brain damage in this condition and provide a tool to monitor brain disease progression and treatment efficacy.

## Material and methods

The study was conducted in compliance with the ethical standard, approved by the local Ethical Committee (62/10), with written informed consent that was obtained from all subjects according to the Declaration of Helsinki.

### Participants

In this retrospective analysis of a prospective monocentric cross-sectional study, part of a larger monocentric framework on the involvement of CNS in FD, 42 patients (mean age = 42.4 ± 12.9, M/F = 16/26) and 49 healthy controls (HC-mean age = 42.3 ± 16.3, M/F = 28/21) from November 2015 to July 2018, were evaluated. All patients had a classic or late-onset pathogenic mutation according to ClinVar (https://www.ncbi.nlm.nih.gov/clinvar/). To be included in the analysis, FD patients should have met the following inclusion criteria: absence of major cerebrovascular events (namely, “stroke-free” patients, meaning no history of acute neurological symptoms or any evidence of acute or lacunar stroke signs at MRI), age > 18 and < 65 years. In a subgroup of 22 FD patients (mean age = 41.2 ± 13.1, M/F = 8/14) clinical and biochemical variables of organ involvement (respectively the FAbry STabilization indEX -FASTEX- score – an index of global clinical stability in FD [15] – and levels of residual α-GalA activity acquired within 1 week from the MRI scan) were retrieved from medical records.

### MRI data acquisition

All subjects underwent an MRI exam using the same 3 T scanner (Trio, Siemens, Germany) equipped with an 8-channel head coil, with the acquisition of the following protocol: a Magnetization Prepared Rapid Acquisition Gradient Echo sequence (MPRAGE; TR = 1900 ms; TE = 3.39 ms; TI = 900 ms; Flip Angle = 9°; voxel size = 1 × 1x1mm^3^; 160 axial slices), a Fluid Attenuated Inversion Recovery sequence (FLAIR; TR = 6000 ms; TE = 404 ms; TI = 2200 ms; voxel size = 1 × 1x1mm^3^; 160 sagittal slices) used for the assessment of white matter hyperintensities (WMH), a single-echo spoiled gradient echo sequence (TR = 16 ms; TE = 7.38 ms; FA = 2°) and a dual-echo flow-compensated spoiled gradient echo sequence (TR = 32 ms; TE_1_ = 7.38 ms and TE_2_ = 22.14 ms; FA = 20°), with the same geometry (voxel size = 0.5 × 0.5 × 1 mm^3^; 160 axial slices) used for the qMRI analysis.

### MRI data analysis

Quantitative R1 and QSM maps were calculated as putative markers of lipid deposition and brain iron respectively, with the latter included in this study to investigate if possible changes in R1 values could be determined by changes in iron concentration [16]. A complete description of the methodology, and all the processing steps performed to compute R1 and QSM maps, is available in previous works [17–19]. Briefly, R1 maps were computed using a variable FA scheme, optimized in order to reduce the acquisition time of the low FA sequence by halving the TR. The biases typically introduced by non-ideal excitation and RF-spoiling were corrected by minimizing the entropy of the R1 distribution as a function of a second-order polynomial FA map according to the method described in [20]. On the other hand, to obtain QSM images the phase of the multi-echo GrE images was unwrapped, and the background phase was removed via the V-SHARP filter [21]. Then, the susceptibility was obtained according to the method described in [22], which effectively removes the streaking artifacts typically arising in the solution of ill-conditioned inverse problems.

The presence of WM lesions in FD patients was investigated, in line with previous studies [4, 6, 23, 24], on FLAIR images using a modified Fazekas score [25]. Furthermore, hyperintense WM lesions were segmented using a semiautomatic approach (Jim 7, Xinapse Systems) and used to fill lesions in T1-weighted volumes for subsequent segmentation steps. Quantitative R1 and QSM maps, as putative markers of lipid deposition and brain iron respectively, were mapped onto the corresponding T1-weighted volumes through affine co-registration of R1 maps using normalized cross-correlation as similarity metric. Quantitative maps were then analyzed at both global (“bulk” analysis) and regional (“voxel-wise” analysis) levels using the hMRI toolbox [26], embedded in the Statistical Parametric Mapping (SPM) framework (https://www.fil.ion.ucl.ac.uk/spm/) and specifically designed for qMRI data processing. The pipeline included: (a) segmentation of structural T1-weighted volumes in different tissue classes (GM, WM and CSF) using SPM12’s unified segmentation algorithm [27], (b) creation of a study-specific template through SPM12’s DARTEL toolbox [28] and (c) application of the estimated deformation fields to normalize tissue class images and quantitative maps to the MNI space. Finally, normalized R1 and susceptibility maps underwent a (d) tissue-weighted smoothing [29] using a 1-mm isotropic full-width at half maximum Gaussian kernel.

For the “bulk” analysis, GM and WM maps resulting from the segmentation procedure were binarized thresholding at 0.50 probability and the resulting masks were used to extract mean R1 and susceptibility values.

### Statistical analysis

Possible differences in terms of age and sex between FD and HC were probed via a two-sample t test and a chi-squared test, respectively.

For the “bulk” analysis, between-group differences in terms of mean GM and WM quantitative metrics were assessed via ANCOVA analyses, adjusting for the effects of age and sex, with a significance level set at *p* < 0.05.

For the voxel-wise analysis, the normalized and smoothed GM and WM maps of both R1 and QSM maps were statistically analyzed to assess local differences between the two groups using a nonparametric approach based on permutations applied to the general linear model [30] via SPM’s Threshold Free Cluster Enhancement (TFCE) toolbox (http://www.neuro.uni-jena.de/tfce), including age and sex as confounding variables. A total number of 5000 permutations were generated, and cluster-like structures were enhanced using the TFCE approach [31], with a significance level set at *p* < 0.05 after correction for multiple comparisons by controlling the family-wise error (FWE) rate.

Finally, qMRI metrics that proved to be different between the two groups were correlated with clinical and biochemical disease markers: the first eigenvariate was extracted from the clusters of significant between-group difference and entered partial correlation analyses with the FASTEX score and residual enzymatic activity, correcting for age and sex.

All statistical analyses were performed by G.P. (10 years of experience) using the Statistical Package for the Social Sciences package (SPSS, Version 23, IBM, Armonk, New York).

## Results

**Fig. 1:**
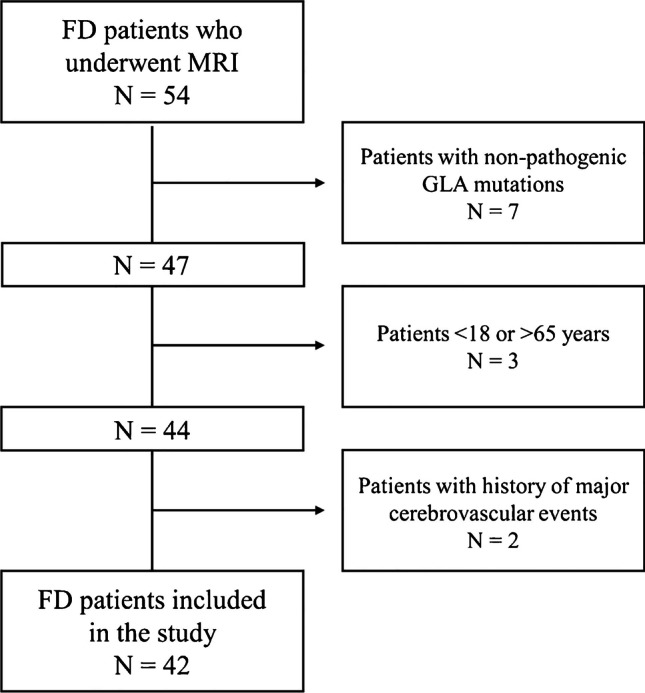
Flowchart showing the number of patients included in the analysis

**Fig. 2:**
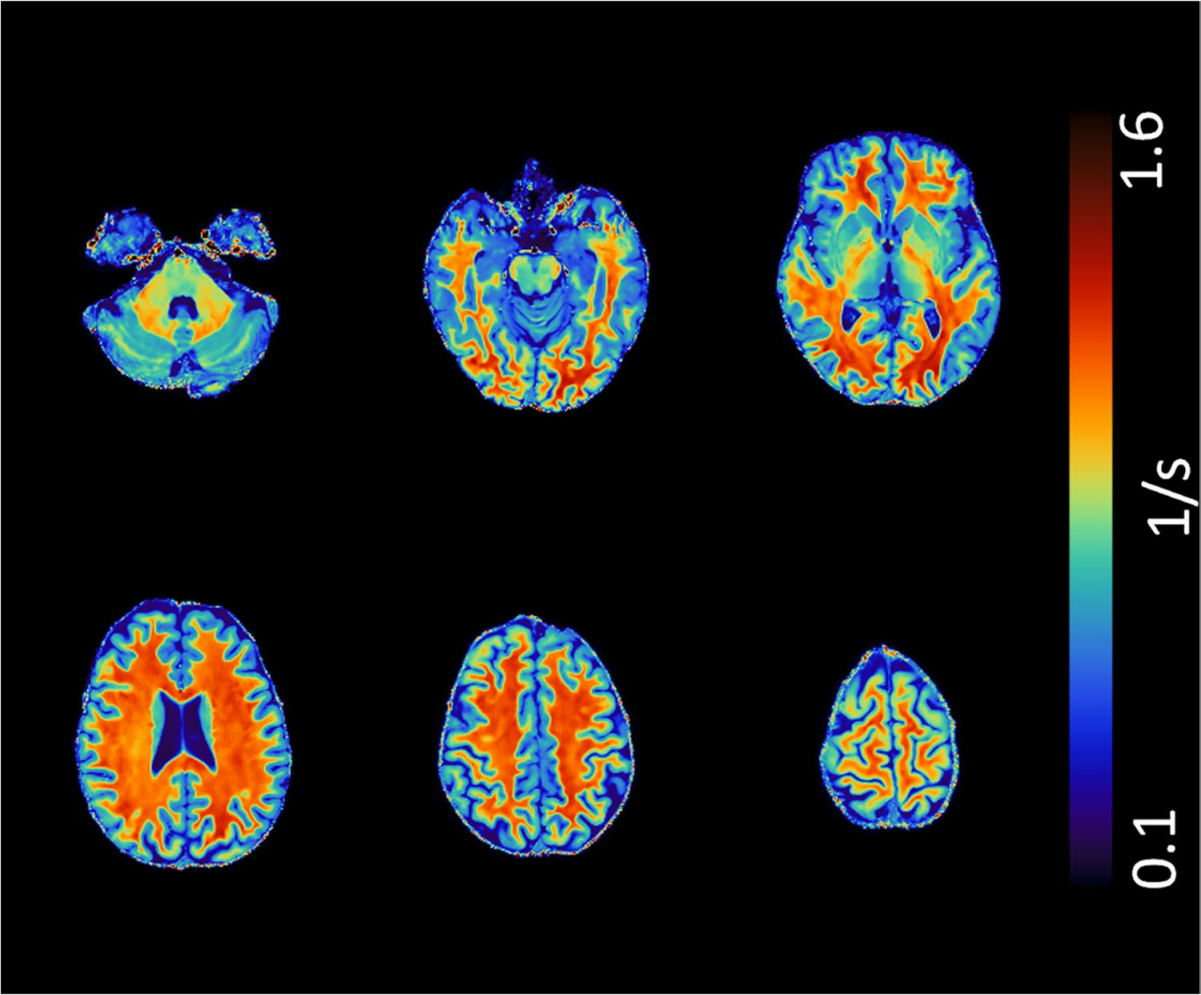
Axial slices of a representative R1 map in a 52-year-old female patient affected by Fabry Disease

**Fig. 3:**
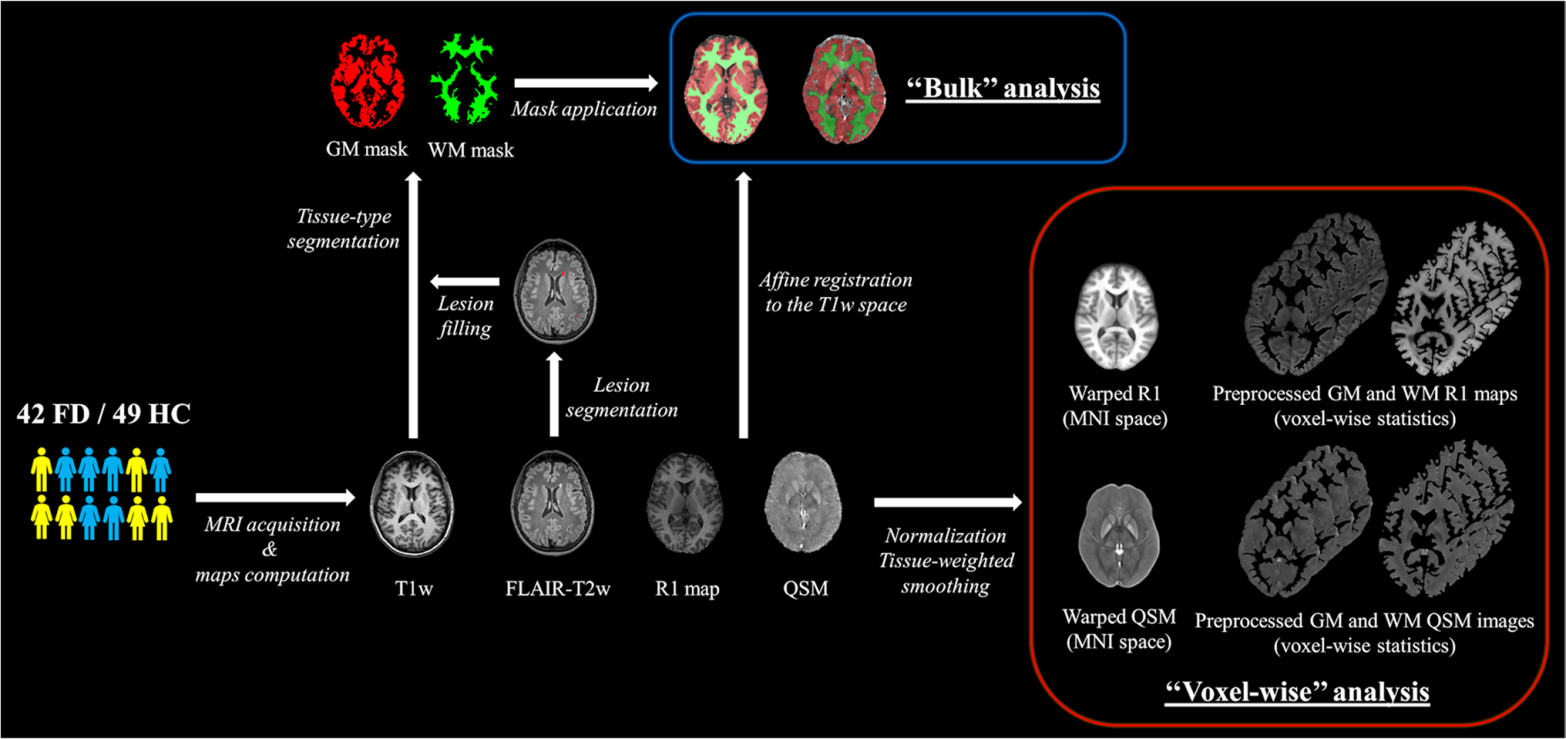
Schematic representation of the main processing steps for the MRI data analysis. GM: gray matter, WM: white matter

Data were obtained from 42 patients (mean age = 42.4 ± 12.9, M/F = 16/26) and 49 healthy controls (HC-mean age = 42.3 ± 16.3, M/F = 28/21) (Fig. 1). A complete list of demographic and clinical information of the studied population is available in Table 1. Representative R1 images are shown in Fig. 2. A graphical representation of the MRI data analysis is shown in Fig. 3.

**Table 1:** Demographic and clinical data of the studied population

	FD	HC	*p*-value
*Age (years)*	42.4 ± 12.9	42.3 ± 16.3	0.98
*Sex (M/F)*	16/26	28/21	0.11
*ERT duration (months)*	35.1 ± 40.6	-	-
Residual α-GalA activity (µmol/L blood/h)^*^	2.44 ± 1.66	-	-
FASTEX score^*^	6 (2 – 13)	-	-
*Fazekas score*	0 (0 – 2)	0 (0 – 1)	-

Data are expressed as mean ± standard deviation, except for sex (M/F ratio) and FASTEX and Fazekas scores (median and range)

^*^available for a subset of 22 FD patients

*FD* Fabry disease, *HC* healthy controls, *ERT* enzyme replacement therapy, *α-GalA* α-galactosidase A, *FASTEX* FAbry STabilization indEX

The FD and HC groups were not different in terms of age (*p* = 0.98) and sex (*p* = 0.11).

At the “bulk” analysis, FD patients showed slightly higher R1 values compared to HC both for the GM (0.70 ± 0.07 vs 0.69 ± 0.05, *p* = 0.08) and WM (1.07 ± 0.10 vs 1.05 ± 0.08, *p* = 0.08) compartments, not reaching the statistical significance threshold. No significant between-group differences emerged in terms of magnetic susceptibility for either GM (5.24 ± 1.69 vs 4.66 ± 1.96, *p* = 0.16) nor WM (-8.94 ± 2.21 vs -8.35 ± 2.51, *p* = 0.29) between FD patients and HC.

**Fig. 4:**
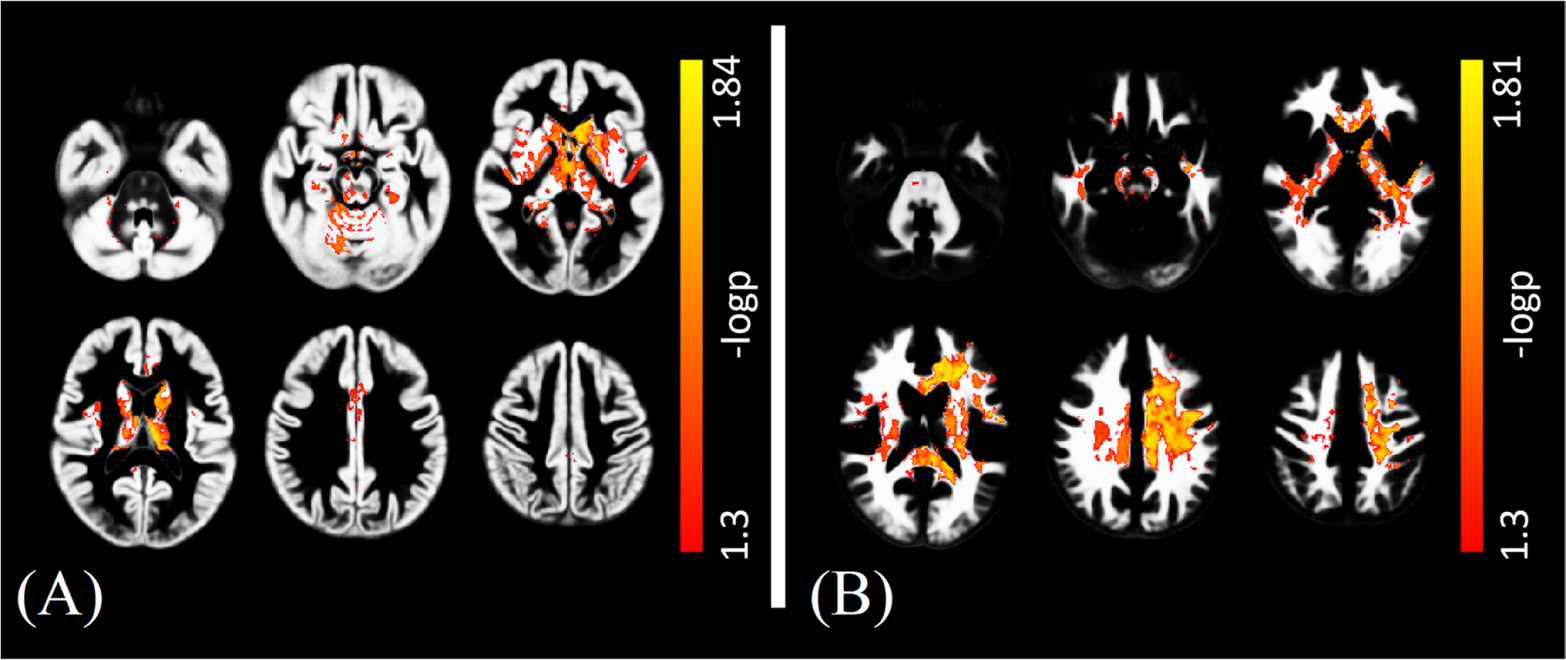
Clusters of significant between-group differences in terms of R1 values for the FD > HC contrast are presented (*in red-yellow*), superimposed on the study-specific GM (left panel, **A**) and WM (right panel, **B**) templates in the MNI space. FD: Fabry Disease; HC: healthy control; GM: gray matter; WM: white matter; MNI: Montreal Neurological Institute

Interestingly, the voxel-wise analysis allowed to show the presence of a widespread R1 increase in FD patients, with several clusters of increased R1 values encompassing various GM structures (*p*_FWE_ = 0.02), including the deep GM nuclei, the insular and the cingulate cortices (Fig. 4A), as well as distributed WM changes (*p*_FWE_ = 0.02) (Fig. 4B). On the other hand, no significant between-group differences emerged when analyzing QSM maps, thus excluding the possible contribution of iron modifications and indirectly confirming that the observed R1 changes were mainly a reflection of lipid accumulation.

**Fig. 5:**
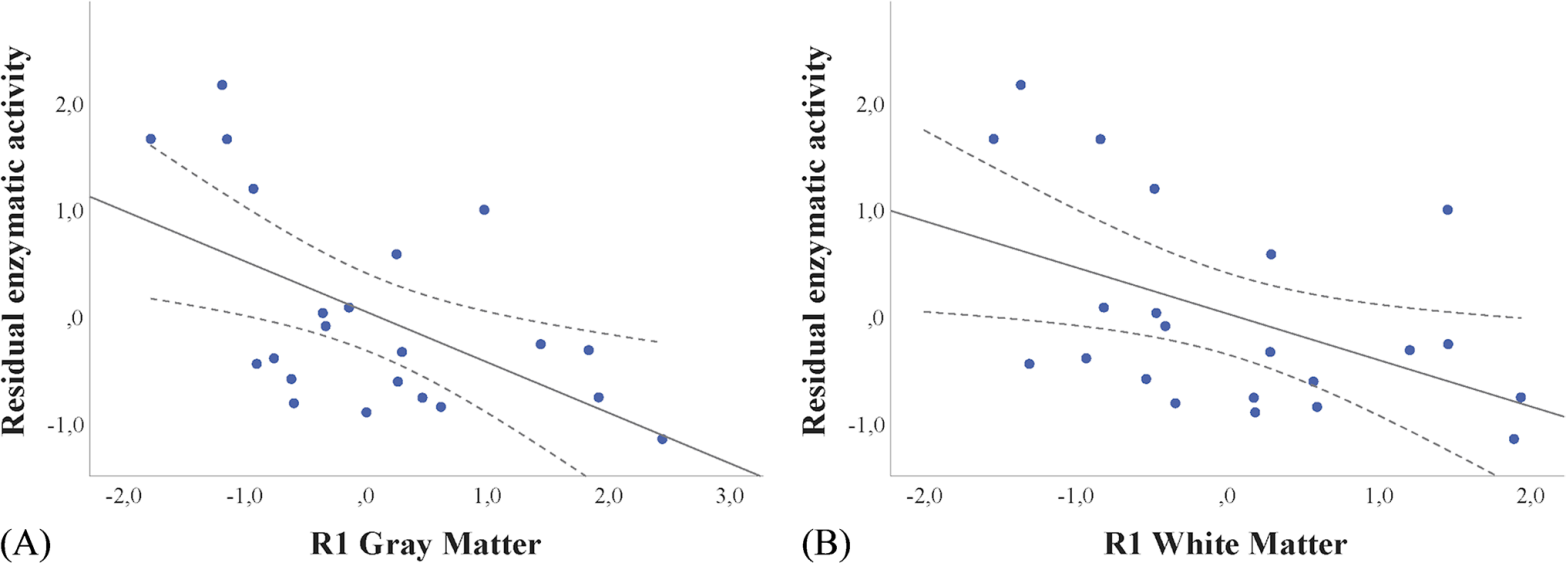
Scatterplot of the correlation between R1 values of the GM (left panel, **A**) and WM (right panel, **B**) and the residual enzymatic activity. GM: gray matter; WM: white matter

When assessing the relationship between clinical, biochemical and MRI variables, a significant negative correlation emerged between residual enzymatic activity levels and clusters of increased R1 values in GM (*r* = -0.57, *p* = 0.008, Fig. 5A) and WM (*r* = -0.49, *p* = 0.03, Fig. 5B). No significant correlations emerged between the clusters of increased R1 values and the FASTEX score (*r* = -0.15, *p* = 0.54 and *r* = -0.22, *p* = 0.35 for GM and WM, respectively).

## Discussion

In this study we expanded the current knowledge on brain involvement in FD, demonstrating the feasibility and clinical relevance of non-invasively assessing cerebral Gb3 accumulation in FD in-vivo using quantitative MRI.

The defective activity of α-GalA leading to Gb3 accumulation is the pathophysiological milestone of FD. This lysosomal accumulation, occurring in different cell types, generates a cascade of events including the activation of inflammatory pathways and ultimately leading to cell damage and death [32]. These Gb3 accumulation-induced phenomena are known to occur in the endothelium and vascular smooth muscle cells of brain vessels, causing vascular remodeling and subsequent cerebrovascular pathology [33]. Also, direct glial and neuronal Gb3 depositions have been hypothesized as possible mechanisms leading to primary neurodegeneration [6]. In particular, pathological studies have demonstrated the presence of lipid accumulation not only at the vessel level, but also in different brain areas, including hippocampus, amygdala, deep GM nuclei and the fronto-parietal cortex [7, 8]. Nevertheless, the nature of CNS involvement in FD is still not completely understood, also because of the inability to demonstrate Gb3 accumulation in the brain with methods other than biopsy, a technique which is relatively widely used for other organs but unfeasible for the assessment of CNS involvement in-vivo [34–36]. Indeed, to date, non-invasive biomarkers allowing for a physiopathology-informed monitoring of CNS damage are still lacking, with brain MRI only used to assess the burden of cerebrovascular pathology in terms of acute accidents and WM hyperintense lesions, which are both late and irreversible events [9].

Our results showed that R1 mapping is sensitive to FD-related brain damage, offering a novel quantitative neuroimaging biomarker of Gb3 accumulation. Indeed, although the correspondence between increased R1 values and lipid deposition is not entirely specific, the correlation between T1 relaxation rates and Gb3 levels has been previously demonstrated in the myocardial tissue [37]. Also, the lack of concurrent significant QSM changes allowed to rule out the role of iron modifications in determining the observed R1 changes, indirectly confirming that these might only be reflecting lipid accumulation. Finally, it should be noticed that a possible contribution of microvascular damage and related myelin changes to the observed R1 increase can be excluded, as WML show a decrease in myelin concentration which should result in lower, rather than increased, R1 values [38].

It is noteworthy to further highlight the analogy between MR relaxometry of the brain and T1 mapping in cardiac imaging, which has been extensively used in the past decade do demonstrate myocardial Gb3 deposition in FD [39] and has taken on an increasingly central role in the clinical management of FD patients [40]. Similarly, R1 mapping of the brain might represent a useful tool to assess FD-related CNS damage since from the early phases of the disease, with relevant implications in terms of prognostic stratification and treatment response monitoring [41].

Interestingly, while the “voxel-wise” analysis allowed to demonstrate the presence of widespread accumulation of Gb3 in the brain of FD patients, the “bulk” analysis failed to show a significant between-group difference in mean R1 values, confirming the known superior sensitivity of voxel-based approaches [42]. Also, while spatially distributed, our findings showed a certain degree of anatomical specificity, potentially reflecting the greater susceptibility to Gb3 accumulation of certain brain regions (e.g., the deep GM nuclei), in line with the few scattered pathological evidences in FD patients [7, 8], as well as the reported distribution of anti-Gb3-Ab reactivity found in an experiment on a mouse model [43].

When investigating the biological and clinical relevance of the observed changes, we found a correlation between clusters of increased R1 values of both GM and WM and the residual enzymatic activity, further strengthening the link between this metric and lipid accumulation and its potential role as a biomarker of disease severity. Nevertheless, we failed to find a significant correlation between R1 changes and the FASTEX score, which might however be explained by the low sensitivity of this scoring system to assess neurological involvement in this setting. Indeed, not only the FASTEX score is intrinsically unbalanced towards non-neurological (mainly cardiac and renal) manifestations, but we also excluded subjects with evidence of major cerebrovascular events [15].

This study comes with some limitations, the first being the absence of direct histological validation. Notwithstanding the abovementioned considerations on the interpretation of our findings, future post-mortem or pre-clinical studies are warranted to confirm the relationship between the observed R1 changes and lipid deposition. Another limitation of our study, mainly stemming from its retrospective nature, is the lack of information regarding the relationship between cerebral R1 values and other novel, more accurate, biochemical markers of disease severity such as Lyso-Gb3 levels [44], as well as with cardiac Gb3 accumulation as assessed via T1 mapping. Future perspective studies are therefore needed to further validate brain R1 changes as an indicator of disease severity against other established biomarkers.

In conclusion, we demonstrated that the non-invasive assessment of cerebral Gb3 accumulation in FD using MRI is both feasible and clinically relevant. R1 mapping might be used as an in-vivo quantitative neuroimaging marker of Gb3 deposition in FD patients, potentially offering a novel tool to monitor tissue damage progression and treatment response.

## Data Availability

Data generated or analyzed during the study are available from the corresponding author by reasonable and appropriate request.

## Abbreviations

α-GalA: α-Galactosidase A
FASTEX: FAbry STabilization indEX
FD: Fabry Disease
Gb3: Globotriaosylceramide
GM: Gray matter
qMRI: Quantitative MRI
QSM: Quantitative Susceptibility Mapping
R1: Longitudinal relaxation rate
WM: White matter
